# Supplementary figures and images for: Multispecies characterization of immature neurons in the mammalian amygdala reveals their expansion in primates
Source: PLoS Biol. 2025 Aug 14;23(8):e3003322. doi: 10.1371/journal.pbio.3003322 (PMC12370197; doi:10.1371/journal.pbio.3003322)

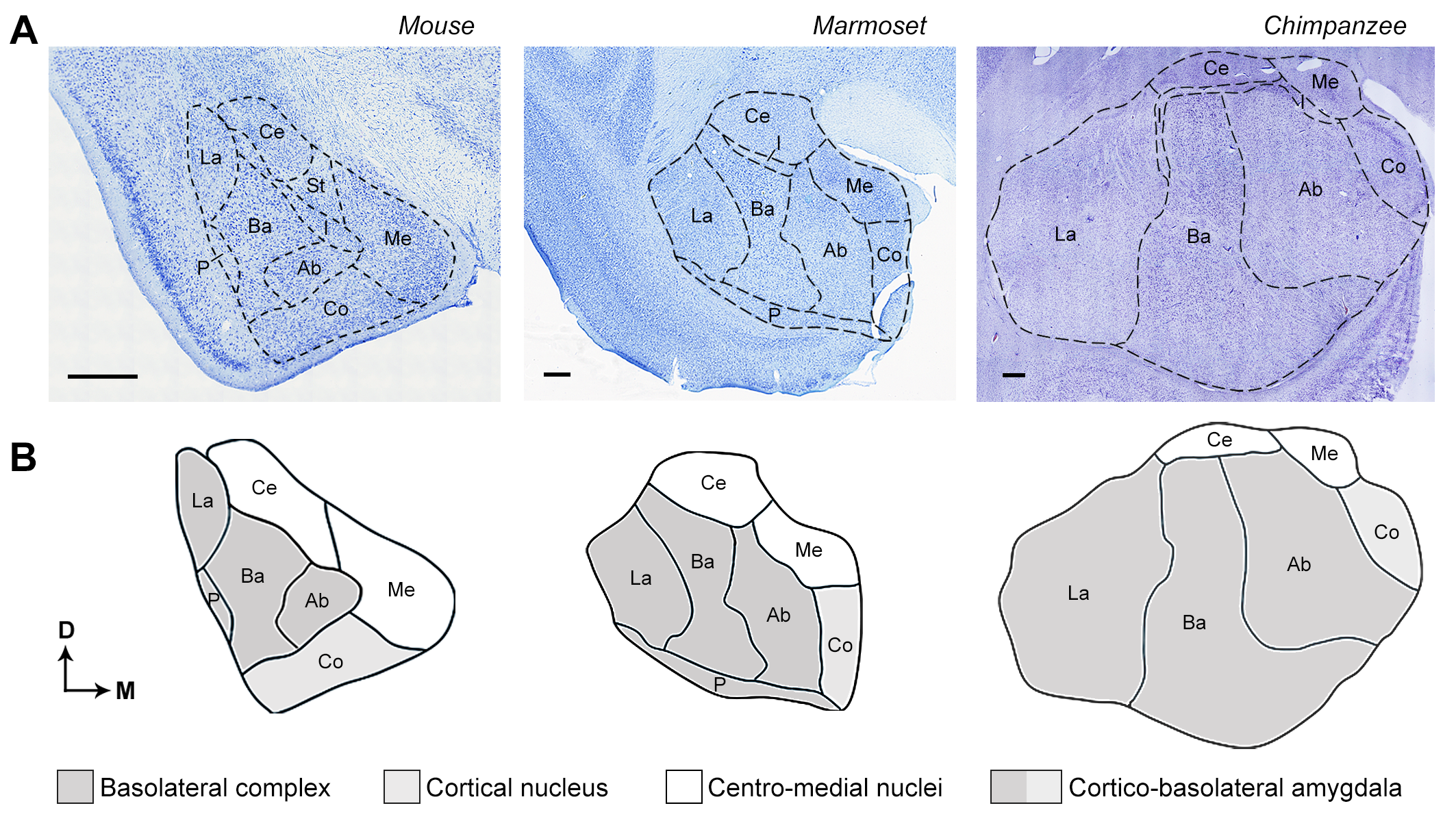

Supplement: S1 Fig — (A) Amygdala subnuclei segmentation was performed on coronal sections stained with toluidine blue; cresyl violet in chimpanzees and matched with: ([7,37,38] mouse; [39] naked mole rat; [40,41] rabbit; [28,42] cat; [43,44] sheep; [45–47] marmoset; [47,48] chimpanzee; [49] horse). Due to heterogeneous and fragmentary literature data concerning the subnuclei segmentation in different species, a simplified delineation and grouping were adopted (here shown in mouse and primates; for all species and different anterior-posterior levels, see Fig 2) to be used for studying the number and distribution of DCX+ cells in all eight species considered here. La, Lateral nucleus; Ba, Basal nucleus; Ab, Accessory Basal nucleus; P, Paralaminar nucleus; Ce, Central nucleus; Me, Medial nucleus; Co, Cortical nucleus; I, Intercalated nuclei; St, Stria terminalis. Scale bars: 500 µm. (TIF) [file pbio.3003322.s001.tif]

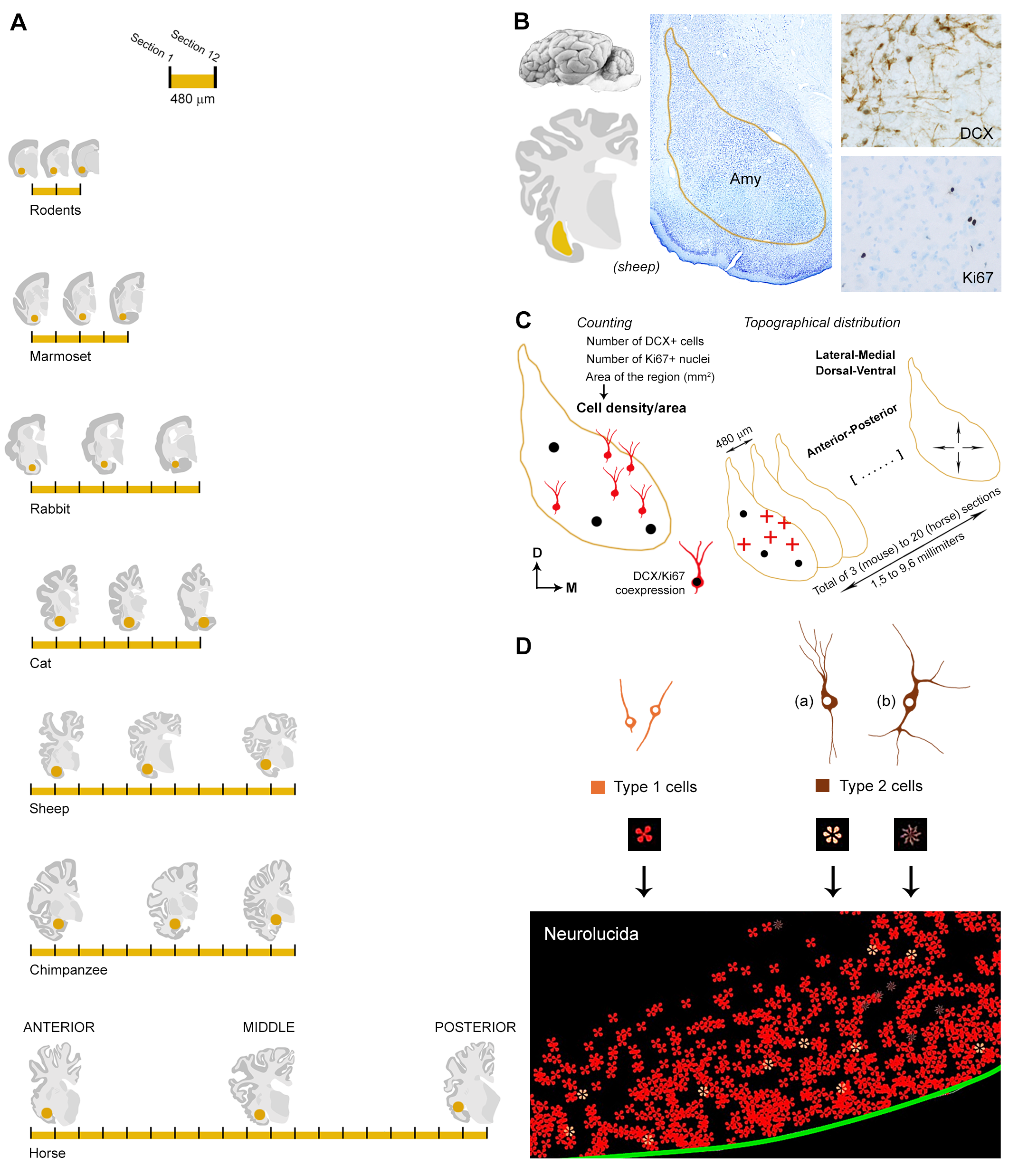

Supplement: S2 Fig — (A) The entire amygdala’s anterior-posterior length of each species (from 1.4 to 9.6 mm) was analyzed immunocytochemically in sections at 480 µm apart (one serial coronal section out of 12; from 3 sections in rodents to 20 sections in horse). (B, C) After establishing amygdala areas through segmentation carried out on serial coronal sections (see Figs 1 and 2), DCX+ cells (red crosses) and Ki67+ nuclei (dividing cells; black dots) were counted with Neurolucida software (example given in D), to obtain cell density/area and cell density/volume of the entire region of interest. The anterior-posterior distribution and the bidimensional spatial distribution (dorsal-ventral and lateral-medial) of immunoreactive elements in single coronal sections were also studied (see Figs 6 and 7). While counting the DCX+ cells, a distinction was made between type 1, type 2a, and type 2b (the latter then considered together as type 2 cells), based on their soma size and dendritic arborization (D; see also Fig 3A). The aim was to obtain comparable cell densities and spatial distributions in all species to investigate phylogenetic variation (and age-related variation), and to check for presence/absence/amount/nature of dividing cells (see text and Table 1). Colors for the two main cell types (yellow and brown) are the same in pie charts of Fig 5C). (TIF) [file pbio.3003322.s002.tif]

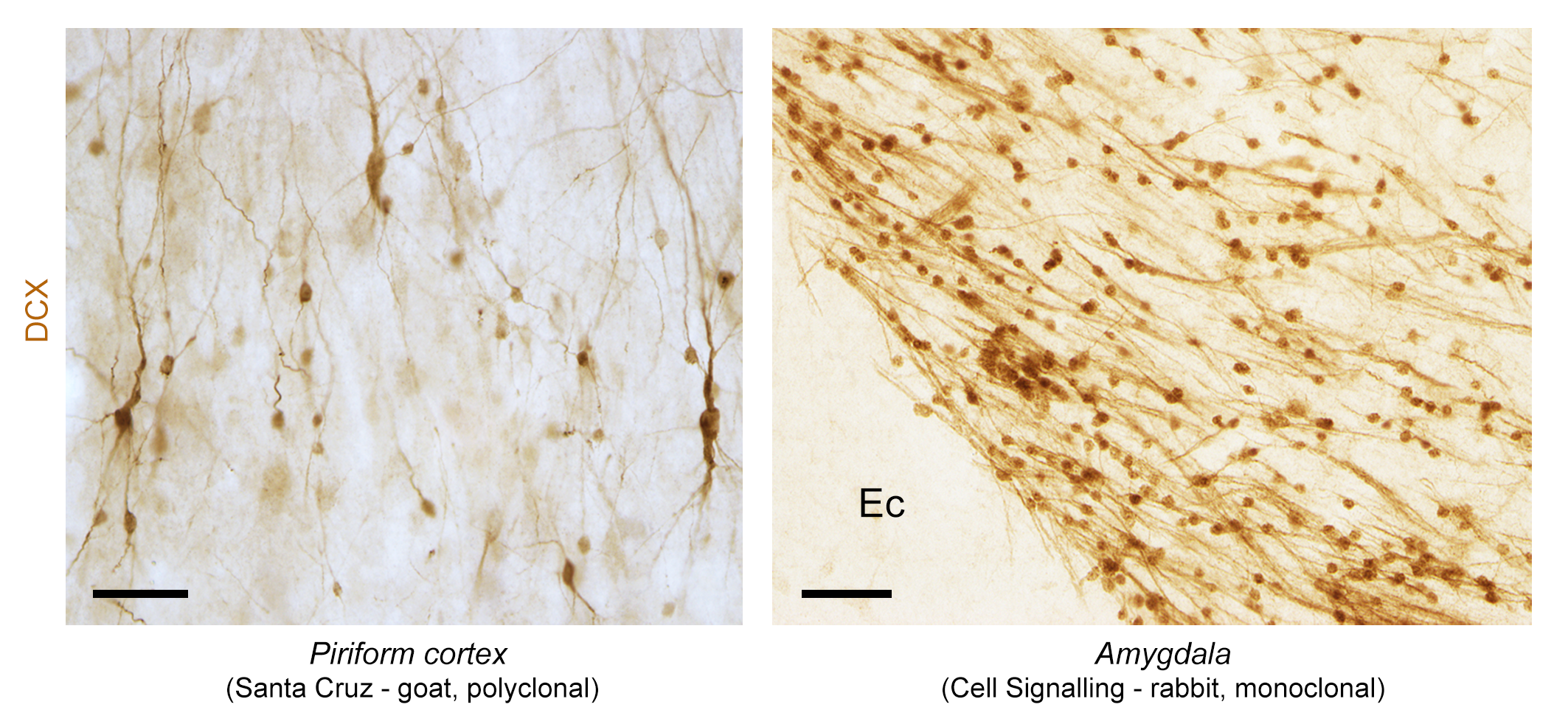

Supplement: S3 Fig — Note the densely packed network of DCX+ cells in the amygdala and the absence of staining in the external capsule (Ec). Scale bars: 50 µm. (TIF) [file pbio.3003322.s003.tif]

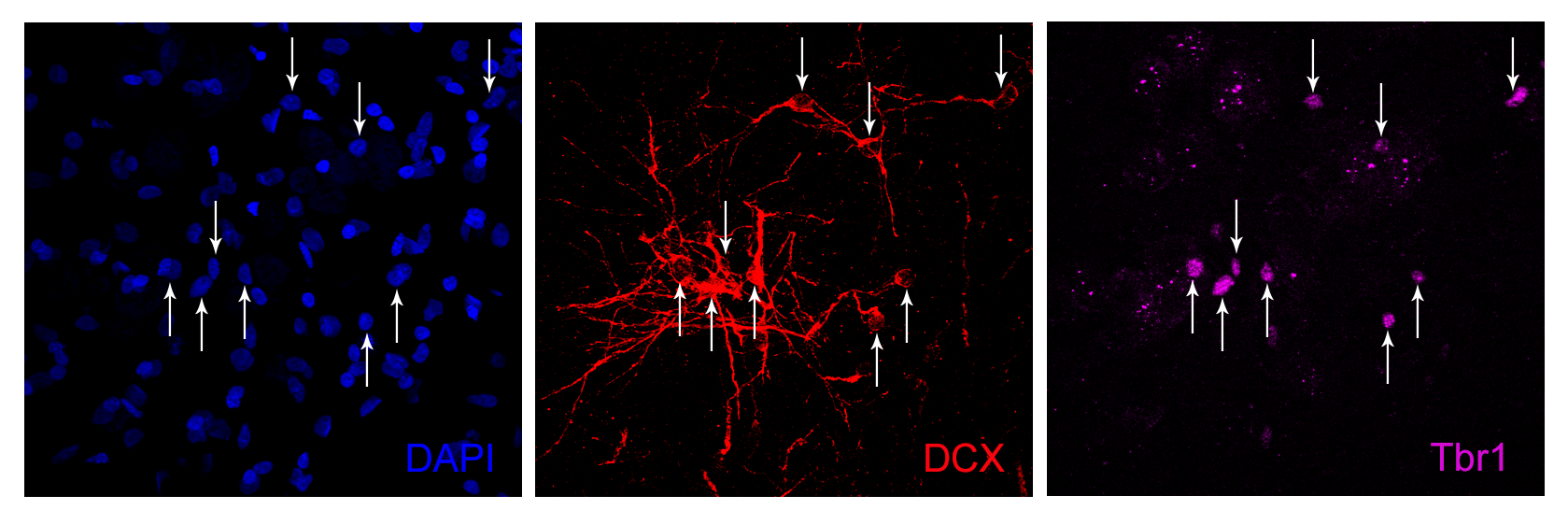

Supplement: S4 Fig — (TIF) [file pbio.3003322.s004.tif]

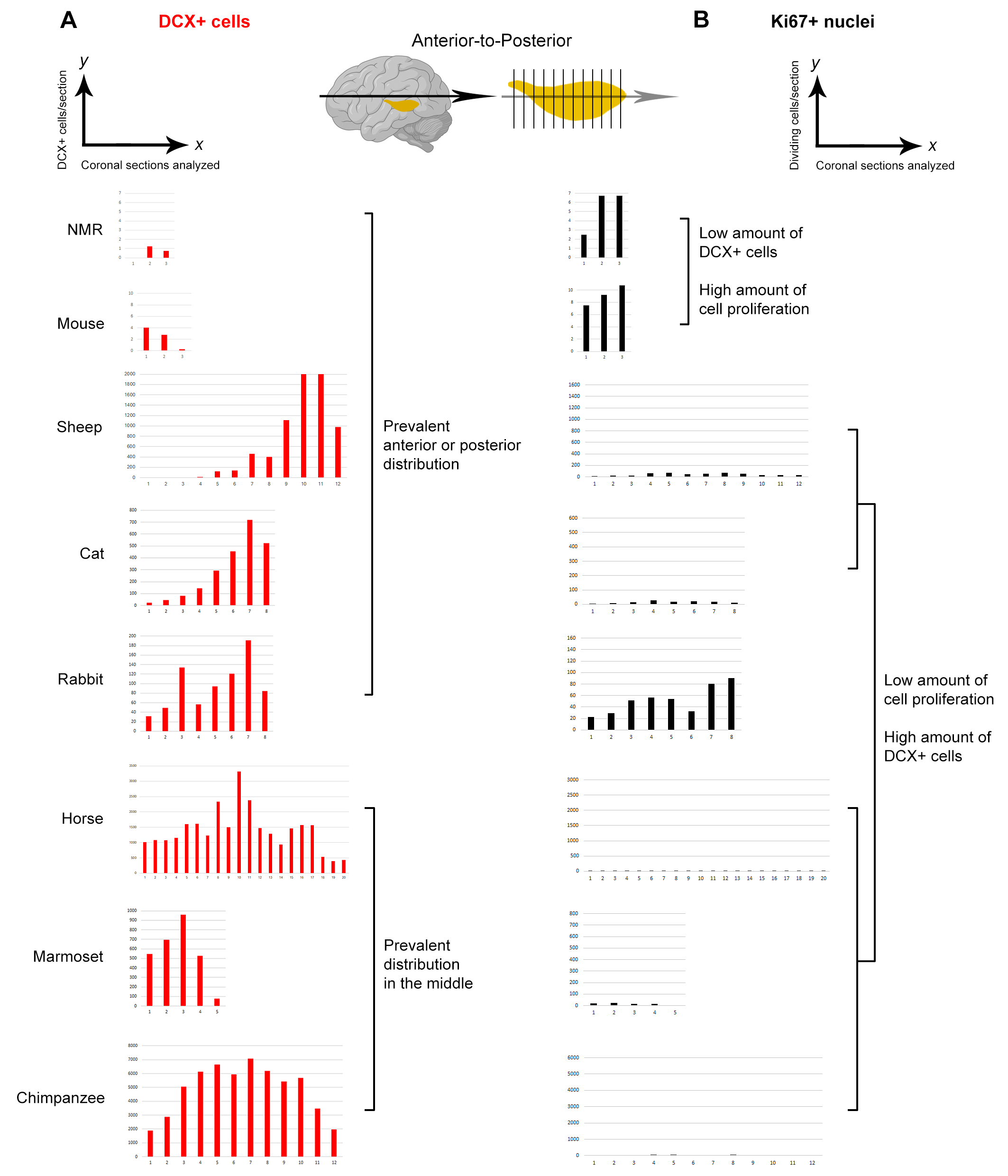

Supplement: S5 Fig — The amount of DCX+ cells (A, density/area; red) and Ki67+ nuclei (B, black) are reported as histograms (y-axis) for each single coronal section of the amygdala counted in each species (from 3 in rodents to 20 in horse, x-axis; see also Fig 6A), in the sequence from the anterior (left) to posterior part (right), thus revealing the pattern of longitudinal spatial distribution of these cell populations. While the distribution of the DCX+ cells is highly heterogeneous among mammals (see also Fig 6A), e.g., far higher in the anterior (mouse), middle (primates), or posterior part (sheep, cat), that of dividing cells is quite homogeneous, especially in gyrencephalic species and marmoset. Animal species are arranged from top to bottom according to increasing DCX+ cell density (mean cell density in the whole amygdala). Tabulated data can be found in S1 Data. Brain icon from https://app.biorender.com/. (TIF) [file pbio.3003322.s005.tif]

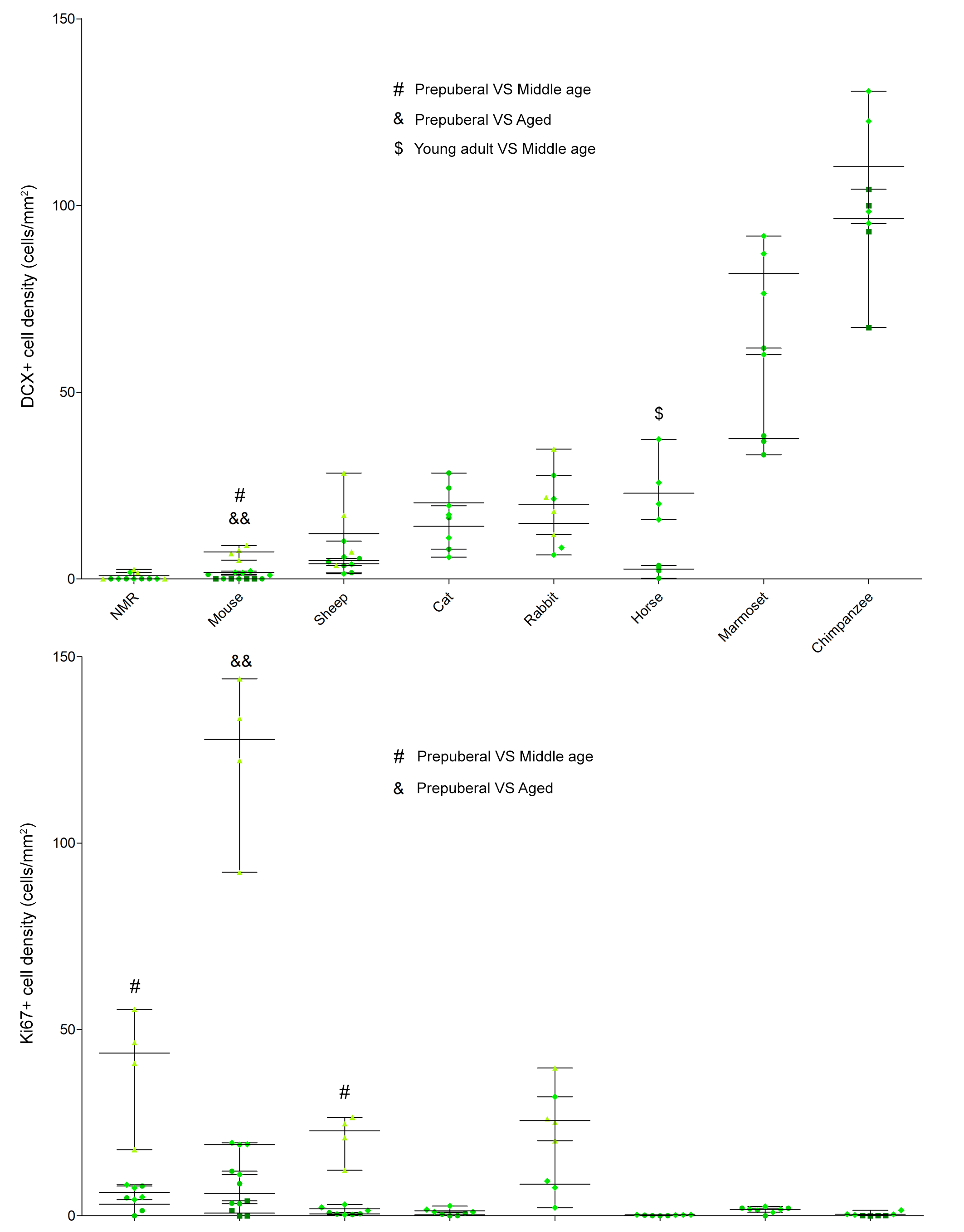

Supplement: S6 Fig — (TIF) [file pbio.3003322.s006.tif]

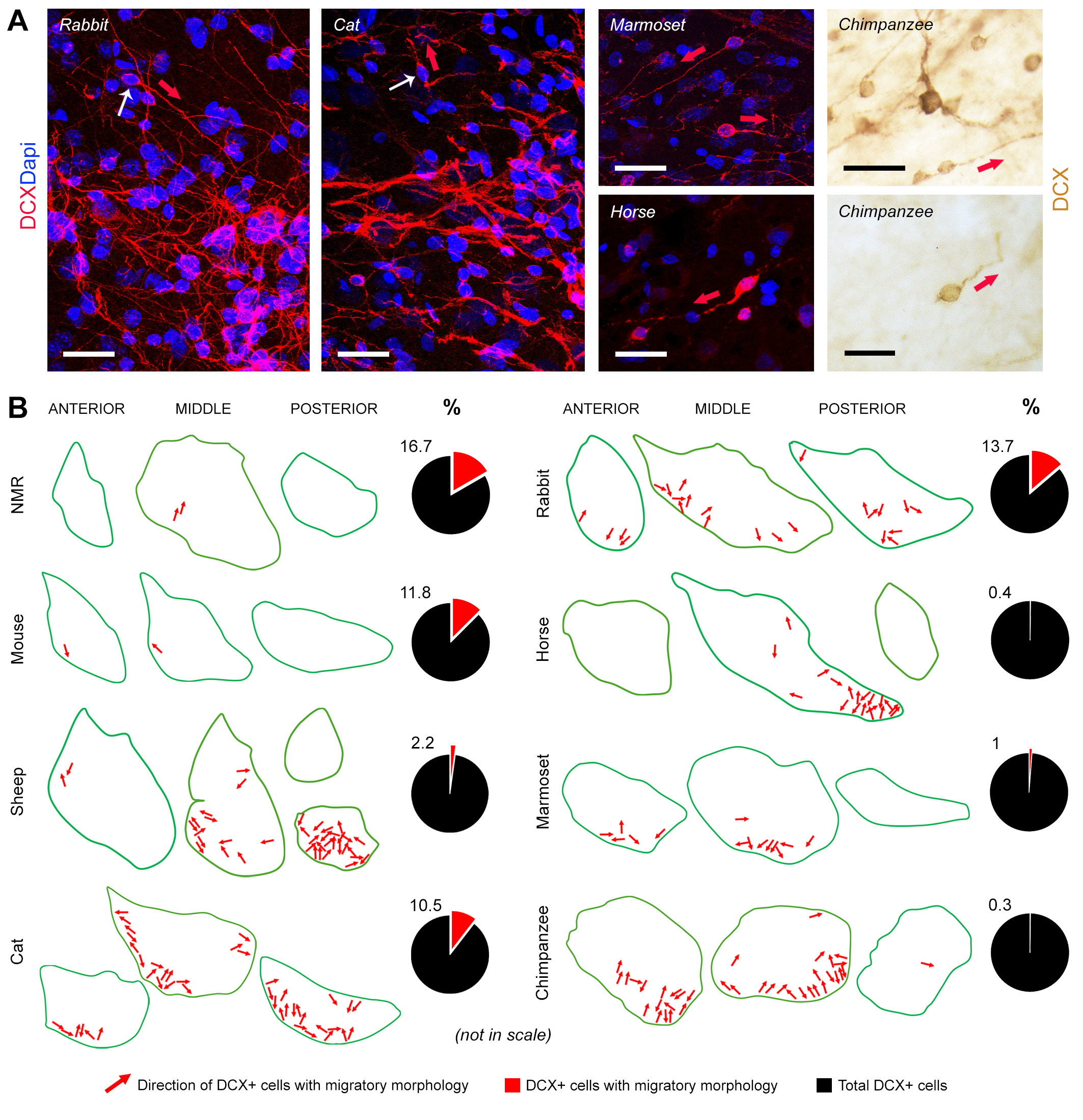

Supplement: S7 Fig — Red arrows indicate the orientation of the leading processes. A, Examples of DCX+ cells with migratory morphology in the amygdala of different species. At low magnification, only some of these cells are detectable (white arrows) out of many immature cells. B, The amount of these cells (percentage with respect to all DCX+ cells in the section), their topographical location in the anterior, middle, and posterior part of the amygdala, as well as the direction of their leading-like process, is shown. Green line, amygdala perimeter. The percentage of migratory-like cells (pie charts) decreases going from rodents to horse and primates, their direction being mostly random in all species considered, thus showing no specific alignment. Scale bars: immunofluorescence, 30 µm; diaminobenzidine staining, 20 µm. (TIF) [file pbio.3003322.s007.tif]
