# Supplementary material for: Multispecies characterization of immature neurons in the mammalian amygdala reveals their expansion in primates
Source: PLoS Biol. 2025 Aug 14;23(8):e3003322. doi: 10.1371/journal.pbio.3003322 (PMC12370197; doi:10.1371/journal.pbio.3003322)
Supplement: S1 Table — (a) Institute of Anatomy – University of Zurich; (b) Department of Comparative Biomedicine and Food Science – University of Padova; (c) National Chimpanzee Brain Resource – USA; (d) Neuroscience Institute Cavalieri Ottolenghi (NICO); (e) INRA research center – Nouzilly, France; (f) School of Biological and Chemical Sciences, Queen Mary University of London, London; (h) George Washington University, Washington DC. PMI: postmortem interval; CA, carotid artery; IC, intra-cardiac; PFA, paraformaldehyde solution. Age groups: PP, Prepuberal; PA, Picric acid; YA, Young adult (in bold since a stage present in all species); MA, middle age; AG, aged; *see text; d, days; m, months; y, years. IC, intra-cardiac; CA, carotid artery. Animal species are arranged from top to bottom according to their increasing brain size. For information regarding ages the Animal Diversity Web ([87]; available at https://animaldiversity.org/) was used. (DOCX) [file pbio.3003322.s008.docx]

**Table S1.** Animals and brain tissues used in this study (4 specimens/age)

| **Species** | **Source** | **Ages** | | **Fixation** | **Fixative** | **PMI** |
| --- | --- | --- | --- | --- | --- | --- |
| *Mouse* | (d) | (PP) 10 d |  | Immersion | 4% PFA | A few minutes |
|  |  | (**YA**) 3 m |  | Perfusion (IC) |  | None |
|  |  | (MA) 9 m |  |  |  |  |
|  |  | (AG) 15 m |  |  |  |  |
| *Naked mole rat* | (f) | (PP) 2 m |  | Immersion |  | A few minutes |
|  |  | (**YA**) 2 y |  | Perfusion (IC) |  | None |
|  |  | (MA) 10 y |  |  |  |  |
| *Marmoset* | (h) | (**YA**) 2.5 y * |  | Immersion | 10% formalin | None |
|  | (a) | (MA) 5-8 y | 5-8 y  5-8 y  5-8 y  6 y |  | 4% PFA+15% PA | 1 hour |
| *Rabbit* | (d) | (PP) 3 m |  | Perfusion | 4% PFA | None |
|  |  | (**YA**) 3 y |  |  |  |  |
| *Cat* | (b) | (**YA**) 1.5 y |  | Immersion | 10% formalin | 1 hour |
|  |  | (MA) 6-7 y | 6 y  7 y  7 y  7 y |  |  |  |
| *Sheep* | (e) | (PP) 4 m |  | Perfusion (CA) | 4% PFA | None |
|  | (b) | (**YA**) 5-6 y | 5 y  6 y  6 y  6 y | Immersion | 10% formalin | 20 minutes |
|  |  | (MA) 8-10 y | 8 y  10 y  10 y  10 y |  |  |  |
| *Chimpanzee* | (c) | (**YA**) 17-24 y | 17 y  18,5 y  19,3  24 | Immersion |  | 14 hours |
|  |  | (AG) 40-48 y | 40 y  41,6 y  44,5 y  48 y |  |  |  |
| *Horse* | (b) | (**YA**) 3-7 y | 3 y  3 y  4 y  7 y | Immersion |  | 1 hour |
|  |  | (MA) 11-16 y | 11 y  15 y  15 y  16 y |  |  |  |

(a) Institute of Anatomy – University of Zurich; (b) Department of Comparative Biomedicine and Food Science – University of Padova; (c) National Chimpanzee Brain Resource – USA; (d) Neuroscience Institute Cavalieri Ottolenghi (NICO); (e) INRA research center – Nouzilly, France; (f) School of Biological & Chemical Sciences, Queen Mary University of London, London; (h) George Washington University, Washington DC. PMI: postmortem interval; CA, carotid artery; IC, intra-cardiac; PFA, paraformaldehyde solution. Age groups: PP, Prepuberal; PA, Picric acid; YA, Young adult (in bold since a stage present in all species); MA, middle age; AG, aged; *see text; d, days; m, months; y, years. IC, intracardiac; CA, carotid artery. Animal species are arranged from top to bottom according to their increasing brain size. For information regarding ages the Animal Diversity Web (87; available at <https://animaldiversity.org/>) was used.
