# Supplementary material for: Multispecies characterization of immature neurons in the mammalian amygdala reveals their expansion in primates
Source: PLoS Biol. 2025 Aug 14;23(8):e3003322. doi: 10.1371/journal.pbio.3003322 (PMC12370197; doi:10.1371/journal.pbio.3003322)
Supplement: S2 Table — (DOCX) [file pbio.3003322.s009.docx]

**Table S2.** Primary antibodies used in this study

| **Antigen** | **Host** | **Type** | **Code (RRID)** | **Raised against** | **Dilution** | **Source** |
| --- | --- | --- | --- | --- | --- | --- |
| DCX | goat | polyclonal | SC8066 (AB_2088494) | Epitope within the last 50 c-terminal amino acids | 1:300-2000 | Santa Cruz Biotechnology |
|  | mouse | monoclonal | SC271390  (AB_10610966) | Amino acids 81-365 mapping at the C-terminus of Doublecortin of human origin | 1:1000 |  |
|  | rabbit |  | 40619  (AB_3696702) | Synthetic peptide corresponding to residues near the carboxy terminus of human doublecortin protein | 1:500 | Cell Signaling |
|  |  | polyclonal | AB18723  (AB_732011) | Synthetic peptide conjugated to KLH derived from within residues 300 to the C-terminus of Human Doublecortin | 1:1000 | Abcam |
|  |  |  | 4604  (AB_561007) | Antigenic sequence surrounds amino acid 350 tyrosine of human doublecortin |  | Cell Signaling |
|  | guinea pig |  | AB2253  (AB_1586992) | Epitope aminoacidic sequence: YLPLSLDDSDSLGDSM |  | Merck Millipore |
| Ki-67 | rabbit |  | AB15580  (AB_443209) | Synthetic peptide | 1:500 | Abcam |
|  | mouse | monoclonal | 550609  (AB_393778) | Human Ki-67 |  | BD Pharmingen |
| PSA-NCAM | mouse |  | MAB5324  (AB_95211) | Viable Meningococcus group B (strain 355) | 1:700 | Merck Millipore |
| NeuN | mouse |  | MAB377  (AB_2298772) | Purified cell nuclei from mouse brain | 1:300 |  |
| Tbr1 | rabbit | polyclonal | AB10554  (AB_10806888) | KLH-conjugated linear peptide corresponding to 18 amino acids from the N-terminal region of mouse T-box brain protein 1 (Tbr1) | 1:1000 |  |
| SOX10 |  |  | HPA068898  (AB_2686054) | Immunogen sequence: PHYTDQPSTSQIAYTSLSLPHYGSAFPSISRPQFDYSDHQPSGPYYGHSG | 1:500 |  |
| Olig2 |  |  | AB9610  (AB_570666) | Recombinant mouse Olig-2 |  |  |
