# Supplementary material for: Multispecies characterization of immature neurons in the mammalian amygdala reveals their expansion in primates
Source: PLoS Biol. 2025 Aug 14;23(8):e3003322. doi: 10.1371/journal.pbio.3003322 (PMC12370197; doi:10.1371/journal.pbio.3003322)
Supplement: S3 Table — La, lateral nucleus; Ba, basal nucleus (*the PL has been included both for species in which it has been previously described and in the others, wherein it is considered as part of the Ba); Ab, accessory basal nucleus; PL, paralaminar nucleus; BLc, basolateral complex (including the PL); Ce, central nucleus; Me, medial nucleus; Ce-Me, centro-medial nucleus; Co, cortical nucleus. Background colors (dark gray, white, and light gray) identify the three amygdala subdivisions shown in Fig 7B. (DOCX) [file pbio.3003322.s010.docx]

**Table S3.** Mean percentages of areas occupied by DCX^+^ immature neurons in the amygdala subnuclei

| **Species** | La | Ba (+PL)* | Ab | **BLc** |  | Ce | Me | **Ce-Me** |  | Co |
| --- | --- | --- | --- | --- | --- | --- | --- | --- | --- | --- |
| *NMR* | 0 | 0.002 | 0.003 | **0.005** |  | 0 | 0 | **0** |  | 0 |
| *Mouse* | 0 | 0.003 | 0 | **0.003** |  | 0 | 0 | **0** |  | 0 |
| *Sheep* | 0.007 | 1.472 | 1.169 | **2.648** |  | 0.015 | 0.015 | **0.030** |  | 0.110 |
| *Cat* | 0.247 | 0.483 | 0 | **0.730** |  | 0.050 | 0.009 | **0.059** |  | 0.760 |
| *Rabbit* | 0.086 | 0.060 | 0.253 | **0.318** |  | 0.014 | 0.104 | **0.059** |  | 0.096 |
| *Horse* | 0.277 | 0.713 | 1.715 | **2.134** |  | 0.330 | 0.990 | **1.155** |  | 4.770 |
| *Marmoset* | 0.049 | 3.150 | 0.656 | **3.636** |  | 0 | 0 | **0** |  | 2.247 |
| *Chimpanzee* | 1.787 | 5.737 | 0.151 | **7.675** |  | 0.040 | 0.060 | **0.100** |  | 0.180 |

La, lateral nucleus; Ba, basal nucleus (*the PL has been included both for species in which it has been previously described and in the others, wherein it is considered as part of the Ba); Ab, accessory basal nucleus; PL, paralaminar nucleus; BLc, basolateral complex (including the PL); Ce, central nucleus; Me, medial nucleus; Ce-Me, centro-medial nucleus; Co, cortical nucleus. Background colors (dark grey, white, and light grey) identify the three amygdala subdivisions shown in Fig 7B.
