# Supplementary material for: Multispecies characterization of immature neurons in the mammalian amygdala reveals their expansion in primates
Source: PLoS Biol. 2025 Aug 14;23(8):e3003322. doi: 10.1371/journal.pbio.3003322 (PMC12370197; doi:10.1371/journal.pbio.3003322)
Supplement: S4 Table — (DOCX) [file pbio.3003322.s011.docx]

**Table S4.** Number of cryostat sections cut in each hemisphere and used for quantitative analyses in the different animal species and ages

| **Species** | **Age** | **N. of sections in the whole hemisphere** | **Whole hemisphere volume analysis** | **N. of sections in the whole amygdala** | **Cell counting**  **and volume analysis**  **in the amygdala** |
| --- | --- | --- | --- | --- | --- |
| *Mouse* | PP | 96 | 8 | 36 | 3 |
|  | YA | 144 | 12 |  |  |
|  | MA | 180 | 15 |  |  |
|  | AG | 144 | 12 |  |  |
| *NMR* | PP | 96 | 8 |  |  |
|  | YA | 120 | 10 |  |  |
|  | MA | 120 | 10 |  |  |
| *Marmoset* | YA | 696 | 58 | 60 | 5 |
|  | MA | 492 | 41 |  |  |
| *Rabbit* | PP | 420 | 35 | 84 | 7 |
|  | YA | 384 | 32 | 96 | 8 |
| *Cat* | YA | 720 | 60 | 96 | 8 |
|  | MA | 516 | 43 | 84 | 7 |
| *Sheep* | PP | 864 | 72 | 132 | 11 |
|  | YA | 1,116 | 93 | 144 | 12 |
|  | MA | 1,152 | 96 | 180 | 15 |
| *Chimpanzee* | YA | 2,304 | 48 | 144 | 12 |
|  | AG | 2,304 | 48 | 144 |  |
| *Horse* | YA | 2,640 | 55 | 240 | 20 |
|  | MA | 2,640 | 55 | 216 | 18 |
