# Supplementary material for: Multispecies characterization of immature neurons in the mammalian amygdala reveals their expansion in primates
Source: PLoS Biol. 2025 Aug 14;23(8):e3003322. doi: 10.1371/journal.pbio.3003322 (PMC12370197; doi:10.1371/journal.pbio.3003322)
Supplement: S5 Table — (DOCX) [file pbio.3003322.s012.docx]

**Table S5.** Estimation of the total number of DCX^+^ cells in the amygdala of different mammals (one hemisphere)

| **Species, age** | **N. of serial sections cut in the entire amygdala** | **N. of sections**  **considered** | **Average N. of DCX^+^ cells in a coronal section**  **of the amygdala** | **Estimation of total DCX^+^ cells in amygdala** |
| --- | --- | --- | --- | --- |
| Mouse PP | 36 | 3 | 14 | **504** |
| Mouse YA |  |  | 2 | **72** |
| Mouse MA |  |  | 0.25 | **9** |
| Mouse AG |  |  | 0.08 | **2.8** |
| NMR PP |  |  | 1 | **36** |
| NMR YA |  |  | 1 | **36** |
| NMR MA |  |  | 0 | **0** |
| Marmoset YA | 60 | 5 | 561 | **33,660** |
| Marmoset MA |  |  | 305 | **18,300** |
| Rabbit PP | 84 | **7** | 113 | **9,492** |
| Rabbit YA | 96 | 8 | 95 | **9,120** |
| Cat YA | 96 | 8 | 285 | **27,360** |
| Cat MA | 84 | 7 | 439 | **36,876** |
| Sheep PP | 132 | 11 | 999 | **131,868** |
| Sheep YA | 144 | 12 | 623 | **89,712** |
| Sheep MA | 180 | 15 | 518 | **93,240** |
| Chimpanzee YA | 144 | 12 | 4,858 | **699,552** |
| Chimpanzee AG | 144 |  | 3,292 | **474,048** |
| Horse YA | 240 | 20 | 1,396 | **335,040** |
| Horse MA | 216 | 18 | 409 | **88,344** |
