# Supplementary material for: Multispecies characterization of immature neurons in the mammalian amygdala reveals their expansion in primates
Source: PLoS Biol. 2025 Aug 14;23(8):e3003322. doi: 10.1371/journal.pbio.3003322 (PMC12370197; doi:10.1371/journal.pbio.3003322)
Supplement: S6 Table — (DOCX) [file pbio.3003322.s013.docx]

**Table S6.** Estimation of the total number of Ki67^+^ cells in the amygdala of different mammals (one hemisphere)

| **Species, age** | **N. of serial sections cut in the entire amygdala** | **N. of sections considered** | **Average N. of Ki67^+^ cells in a coronal section**  **of the amygdala** | **Estimation of total Ki67^+^ cells in amygdala** |
| --- | --- | --- | --- | --- |
| Mouse PP | 36 | 3 | 261 | **9,396** |
| Mouse YA |  |  | 9 | **324** |
| Mouse MA |  |  | 3 | **108** |
| Mouse AG |  |  | 2 | **72** |
| NMR PP |  |  | 16 | **576** |
| NMR YA |  |  | 5 | **180** |
| NMR MA |  |  | 1 | **36** |
| Marmoset YA | 60 | 5 | 15 | **900** |
| Marmoset MA |  |  | 8 | **480** |
| Rabbit PP | 84 | 7 | 95 | **7,980** |
| Rabbit YA | 96 | 8 | 52 | **4,992** |
| Cat YA | 96 | 8 | 15 | **1,440** |
| Cat MA | 84 | 7 | 4 | **336** |
| Sheep PP | 132 | 11 | 443 | **58,476** |
| Sheep YA | 144 | 12 | 40 | **5,760** |
| Sheep MA | 180 | 15 | 19 | **3,420** |
| Chimpanzee YA | 144 | 12 | 27 | **3,888** |
| Chimpanzee AG | 144 |  | 4 | **576** |
| Horse YA | 240 | 18 | 12 | **2,880** |
| Horse MA | 216 | 20 | 5 | **1,080** |
