# Supplementary material for: Multispecies characterization of immature neurons in the mammalian amygdala reveals their expansion in primates
Source: PLoS Biol. 2025 Aug 14;23(8):e3003322. doi: 10.1371/journal.pbio.3003322 (PMC12370197; doi:10.1371/journal.pbio.3003322)
Supplement: Attachment — Submitted filename: ReviewerComments-Revision-PlosBiol.pdf [file pbio.3003322.s017.pdf]

Dear Editor,

Here following you will find our answers to the Reviewer's comments and all changes we made in the Manuscript, Figures, Tables, and Supplementary material.

As requested, we revised the English of the manuscript (Reviewer 1), and we performed new analyses concerning the amygdala orientation and subnuclei segmentation/representation in all species studied (Reviewer 2), as well as the occurrence and orientation of DCX+ cells with migratory morphology (Reviewer 3).

Overall, six Figures (out of nine) were changed/reorganized, and six new Figures were added in the Supplementary material. We thank the Reviewers for constructive comments/suggestions, and we think that the manuscript is now substantially improved.

Reviewer #1:

This study by Ghibaudi et al. builds upon previous work by the research group of Luca Bonfanti, dealing with the characterization of so-called "immature neurons" in different brain regions and different species. "Immature neurons" in this context stand for cells that fulfill the criteria for immature neurons as they occur during neurogenesis, but are found in brain regions in which no de novo neurogenesis from resident or distant precursor cells occurs. The group has developed clear criteria for this population of cells and these criteria are also applied in this new study.
Generally, this research has already added an important and very interesting facet to the discussion of neurogenesis in the adult brain, highlighting that the presumed functional benefits of immature neurons are not dependent on stem cell proliferation and the complete progression through all stages of neurogenesis. The focus of the present descriptive study is on these cells in the amygdala of different species, finding that these cells are more prevalent in primates than in other species. While immature cells, e.g. in the form of doublecortin (DCX) expressing cells, have been described for the amygdala before, and there have been, albeit highly controversial, reports on complete adult neurogenesis in the amygdala, the subject has never been studied thoroughly and especially not in the light of the insight that Bonfanti and colleagues have provided for this intriguing population of neuronal cells.
The submitted manuscript now offers a very detailed and in-depth description of immature neurons across different species. The presentation of the results is excellent and the figures are very appealing. The depth of description is outstanding. It sets and passes neuroanatomical standards rarely seen in publications today. The authors dare to be descriptive, which is also rare, but occasionally necessary. The paper lays a thorough foundation for other studies that might make attempts to address these cells from a functional or molecular perspective.
The key bird's-eye-conclusion from the analysis is that considerable differences exist between different species, both with respect to the distribution of the doublechortin-positive and of proliferating cells in the amygdala. The authors make a very strong case for their

conclusion that the described cells in the amygdala are indeed neurons arrested in maturation or immature neurons, extending their past description for the neocortex to basal brain structures. The most interesting finding is that it seems that this particular population of cells with its implications for plasticity are more abundant in primates than in other mammals.

ANSWER: we thank the Reviewer for his/her thorough analysis of the entire manuscript and for overall appreciation of our work.

The data as they are presented raise little questions or concerns and the general conclusion is easy to follow. The only major criticism is that the text is a bit too rich in words and often not very clear. Already the title appears somewhat convoluted. The text could be tightened without too much effort and the text reworked to obtain a clearer narrative and flow. It could be more to the point. The abstract could be more detailed and more informative. The discussion of how the concept of immature neurons fits into ideas of general brain plasticity could be much clearer.

ANSWER: we edited our text, both manuscript and abstract, aiming at a clearer narrative and flow

Reviewer #2:

This is a very interesting manuscript on the presence of immature neurons (INs), identified by immunoreactivity for cytoskeletal protein doublecortin (DCX), in the amygdala across eight species of mammals, including species with a small, lysencephalic brain (like the mouse, the naked mole rat and the rabbit) to species with a larger brain and different degrees of gyrencephaly (cat, sheep, horse, chimpanzee). They also include the marmoset, a small primate with a mostly lysencephalic brain. The authors undertook a systematic investigation across these eight species, from prepuberal to aged animals, in order to assess phylogenetic variation in the presence and abundance of INs, and compare the expression of DCX with markers of cell proliferation, such as Ki67, and markers of neuronal immaturity (adhesion molecule PSA-NCAM), neuronal differentiation (NeuN), and oligodendroglia (Sox10, Olig2) to evaluate the nature of the DCX+ cells. They find expression of Ki67 in scattered cells of the amygdala adult animals (more in rodents than in primates), but with a distribution pattern different from that of DCX+ cells, and they never find coexpression of DCX and Ki67, but they see coexpression of Ki67 and oligo makers, concluding that proliferation in adult amygdala is basically related to production of glial cells, but not neurons. Based on coexpression of DCX and PSA-NCAM and less frequently DCX and NeuN, the authors conclude that the DCX mostly include immature neurons at different degrees of maturation. They propose that these DCX cells represent a reservoir of non-dividing immature neurons of the amygdala (as found in the neocortex), which become gradually more abundant and stable with age in animals with large gyrencephalic brains and, specially, in primates (both lysencephalic and

gyrencephalic). These INs with 'arrested maturation' appear to represent a source of plasticity in animals with high social cognition abilities and learning demands. This type of studies are highly relevant as they contribute to a better understanding of brain plasticity (including that in the human brain) and here the authors present high quality material supporting their major findings.

ANSWER: we thank the Reviewer for his/her thorough analysis of the entire manuscript and for overall appreciation of our work.

However, I have the following concerns and provide some comments and suggestions that may help to improve the impact of the study.

Major concerns:
1. Topography versus topology: In the results the authors provide a 'topographical' distribution of the DCX+ cells and compare it across species. However, the use of topographical coordinates is highly inadequate for comparing across species with different degrees of brain growth and gyrencephaly. For example, what is lateral in the amygdala of a mouse, it becomes ventral or even ventromedial in a monkey. Thus, I suggest to change to 'topological coordinates', which are the natural coordinates that relate to the internal axes. This will provide a better understanding of the comparison of the DCX across species. As an example, in page 20 and figure 5 legend, the authors say that DCX+ cells tend to locate in 'ventral' or 'ventromedial' positions. In which species? These are topographic terms and the position clearly change depending on the species brain being analyzed. It would be more useful to use some clear landmark to refer to the position, such as if the cells are
adjacent to the amygdalar capsule (sometimes referred to as the external capsule).
Another possibility is to start talking with precision about the amygdala subdivisions where these cells are located (see next point), as when looking at the images it appears that there is a trend fo the DCX cells to be found in or around the so-called paralaminar nucleus (as seen in Fig. 4C, in topographical terms this nucleus is lateral in mouse, but ventral in marmoset and chimpanzee). If the authors do not like the term paralaminar (although they represent it in the Fig. 4C), they can stay with the Ba or BLc (mentioning, for example, that the cells are mostly at the external border, next to the amydalar capsule), and at least readers will know better where the cells locate.

ANSWER:
- We agree with the Reviewer that our representation was not completely clear. At the same time, some of this information was already present in a supplementary figure (Fig. S1 of the original manuscript; we fear that such figure was not available to the Reviewer, because also another Reviewer missed a Table).
However, we admit that the results were not enough explicit. Particularly, the sentence indicated by the Reviewer in page 20 and figure 5 legend: “DCX+ cells tend to locate in 'ventral' or 'ventromedial' positions” sounds as a generalization and, as such, it is wrong. As a matter

of fact, it was only referred to the image of that figure (marmoset amygdala, in D), in which the cells are mostly in ventral-medial position. A similar sentence and generalization were also present in the text. Now, these sentences have been changed.
- To make the comparative anatomy of the amygdala clearer in the manuscript, we split the above-mentioned Figure (now Figure 2 in the text and Fig. S1 in the Supplementary material); in Fig 2, on the left, we introduced the topological representation of the amygdala of all species considered (by combining our histologically stained sections with atlases and other reports) taking into account its orientation with respect to internal axes of the brain, as well as the surrounding anatomical structures. The subdivision in subnuclei (represented on the right) was re-examined, further checking their orientation and subdivision (see point below and the new supplementary Figure S1). All these aspects are now addressed by new Figures and explained/expanded in the figure legends and in the manuscript text.

The overall location of the DCX+ cells within the amygdala and its subdivision was not affected, the vast majority of the INs being consistently located in the Basolateral complex (and, to a lesser extent, in the cortical nucleus, in horse and marmoset). As explained in the next point, we maintained a simplified (though revised) subdivision common to all species considered, to establish the association of the DCX+ cells with the main subdivisions/subnuclei of the amygdala.

2. Amygdala subdivisions: These are represented in Fig. 4, although the authors do not refer to them until too late in the Results. For example, they do not mention these in Figure 5, or the description in the text related to Fig. 5. I think that amygdalar subdivision should be present in the description of the results from the very beginning, and the images shown in Figure 4 are a start point.
However, the delineation of the nuclei represented in Fig. 4C require revision. For example, in mouse, Ce is including the intercalated cells, La is including part of the dorsal endopirifom nucleus, Me is including part of the intercalated cells and the intraamygdaloid subnucleus of BST, but its ventral part has erroneously been assigned to Co. Please use the Paxinos atlas to improve delimitation of nuclei. The same is true for the primate amygdala nuclei (specially problematic is the delineation of the medial amygdala). The authors can refer to the study by Barger et al., 2012, in J. Comp. Neurol (JCN 520:3035-3054; one of the authors is co-author in the present manuscript), and for the medial amygdala the paper by Ghashghaei and Barbas, 2002, Neuroscience Vol. 115, No. 4, pp. 1261-1279).

ANSWER: we agree that amygdalar subdivision should be present earlier in the description of the results (now Fig. 2). We refined the amygdala subnuclei subdivisions in mouse, marmoset and chimpanzee by further checking on atlases and research reports (see revised figure S1). Yet, a very precise subdivision is not available in all species considered (e.g., horse). As a matter of fact, the knowledge of amygdala subdivisions is fragmentary and non-homogeneous in some species. In addition, it must be said that when counting the DCX+ cells on Neurolucida it is not possible to maintain a precise subdivision of small nuclei, especially

in gyrencephalic brains (in some sections of chimpanzee amygdala there are up to 10,000 DCX+ cells). For this reason, in view of placing the DCX+ cells in different parts of the amygdala we adopted a simplified subdivision, by aggregating some nuclei (the correspondence between the two levels of precision is now reported in Fig. S1), with a scheme that might be shared by all species (now reported in Fig. 2, which was revised in some parts following the reviewer's suggestions).
Nevertheless, it appears clear that the vast majority of the DCX+ cells are in the Basolateral complex, whenever it was located or oriented in the different species (mostly ventral-medial in primates, or lateral in rodents). Following the revised delineation, we analyzed again the % of DCX+ cells in each nuclei/species (Table S3 has been edited with the new data). Percentages did not significantly change from the previous version of the manuscript, aside from the % of DCX+ cells in the cortical nucleus of chimpanzee and marmoset (which decreased and increased respectively).
The suggested References were used and cited (we thank the Reviewer for this indication).

Other comments:
3. Fig.1: What means the pie-chart in A (top left)?

ANSWER: this is the mammal species pie chart, illustrating the distribution of extant and recently extinct mammal species across orders (based on Wilson, D. E. and D. M. Reeder eds. 2005. Mammal Species of the World a taxonomic and geographic reference third edition. Johns Hopkins University Press, Baltimore). We indicate the six orders, and the eight species considered in the study. The explanation has been inserted in the Figure legend.

4. Results (page 14 of PDF), second paragraph, line 3: Regarding DCX cells 'being absent in all coronal section', do you mean being scarce, instead of absent? If you say absent, it would be incoherent with saying that there were 'only a few, scattered DCX+ cells', as stated in this same sentence and shown in Fig. 2.

ANSWER: YES, this was a spelling mistake: it was intended that the DCX cells in rodents were absent in some coronal sections (namely, not present in all sections), because they were overall scarce. The sentence has been corrected.

5. Fig. 2: Please explain the fluorescent staining better: in red DCX, in blue DAPI? (the same happens in other figures).

ANSWER: the nature of the staining has been fully explained in the figure legend.

Also, what are you pointing with the arrowheads: a specific morphological subtype of DCX+ cell, or just examples of DCX+ cells?

ANSWER: Arrowheads indicate some type 2b cells. Indeed, it was not indicated in the figure legend. Now it has been added.

In addition, the amygdala is extremely complex, what part of the amygdala are you showing here? See comment above about amygdala subdivisions.

ANSWER: DCX+ cells were mostly located in the basolateral complex, while Ki67+ cells were randomly widespread in the entire amygdala (see Figure 5D, as an example). Hence, the photographs of DCX+ cells always refer to different parts of the Basolateral complex (this has been now specified in the figure legend).

Moreover, it would be relevant to mention somewhere that, from a developmental point of view, the amygdala of all amniotes contains a pallial part (including the basal nuclear complex and cortical areas, rich in glutamatergic neurons), a subpallial part (including the central nucleus, rich in GABAergic neurons), and another division (the medial nucleus) that contains a mix of GABAergic and glutamatergic neurons with different embryonic origins in the pallium, subpallium and the telencephalon-opto-hypothalamic domain. It is interesting to know the location of the DCX cells and the Ki67 cells with respect to these major subdivisions.

- We thank the Reviewer for suggesting the different origin and location of glutamatergic and GABAergic neurons in the subdivision of amygdala. Indeed, the DCX+ cells (which mostly co-express the marker for pallial origin Tbr1) are located almost exclusively within the basolateral complex (and, to a lesser extent, the Co) namely a region largely hosting glutamatergic neurons. The reference by Medina et al., 2023, dealing with the different origin of neuronal population in the amygdala of amniotes, was added, and this aspect was mentioned in the Discussion.

6. Results (page 16 of PDF), regarding 'their glutamatergic identity was confirmed by co-expression with the excitatory neuron transcription factor T-box brain 1 (Tbr1; 38, 39; Fig. 3A': To really appreciate coexpression, it would be important to show the separate channels (for each marker), in addition to the merged image.

ANSWER: Actually, the picture in Fig. 3 is small, and it is not easy to appreciate co-expression. As suggested, we also showed the separate channels of each marker in a Supplementary figure (Fig S4).

7. Fig 3: The red arrowheads in A (in left image) are very difficult to see. I suggest to change the color or the tone to improve visibility.

ANSWER: A change has been made in the figure to improve visibility, by creating white and red arrowheads (white arrowheads have been avoided, since white arrows are used to indicate Ki67+ nuclei).

8. Results (page 20 of PDF, line 1), regarding 'In Fig. 5A are reported the results for quantifications in young adult animals...': suggest to move 'are reported' to after 'animals'

ANSWER: The sentence has been modified as suggested. 'In Fig. 5A the results for quantifications in young adult animals are reported...'

9. Fig 7: In addition to the relative area occupied by the DCX+ cells, another value that could be interesting to show is the density of the DCX+ per mm2 in that area. For example, cat and rabbit show similar area percentage occupied by DCX+ cells, but they appear to be very different in density of DCX+ cells in the paralaminar nucleus.

ANSWER: As reported above, and explained in the text, the degree of knowledge of amygdala subdivisions highly differs among different species, and when counting the DCX+ cells on Neurolucida it is not possible to maintain a precise subdivision of nuclei, especially in some gyrencephalic brains (for instance, in horse we analysed 20 coronal sections but there are no accurate atlas defining the nuclei subdivisions at all anterior-posterior levels of the amygdala). These are the reasons why we adopted a simplified subdivision (yet common to all species) across species.
Considering the above-mentioned difficulties and the main goal of our study (to identify possible phylogenetic variation between species and the association between the scINs and the cortico-basolateral amygdala), we calculated the percentages based on the analysis of three levels (anterior, middle, posterior) in which the main subdivisions (BLc, cortical nucleus, centro-medial complex) are identifiable with reasonable precision. Such an analysis, with cell densities, cannot be performed on Neurolucida (the cell densities were counted within the entire amygdala perimeter), but exporting the images on Image-J.

At the end of the Figure legend, the authors say that 'Animal species are arranged from top to bottom according to increasing DCX+ cell density ', but from the images this is not clear in the case of rabbit versus cat, or even with respect to sheep. Both cat and sheep show higher densities than rabbit.

ANSWER: the increased density from top to bottom is referred to the final average results of quantifications considering all animals (data reported in graphics of Fig 5). Of course, such median value does not correspond to single coronal sections. In Fig 7 are reported coronal sections taken at anterior, middle, and posterior parts of the amygdala (and that are representative of those levels) but the distribution of the DCX+ cells markedly vary at different anterior-posterior levels in different species (as can be seen in the line plot of Fig 6A and in

histograms of Fig S5). To avoid confusion, we further explained this aspect in the figure legend.
NOTE: Throughout the manuscript, the eight animal species considered here are listed according to increasing brain size in the first part (before DCX cell counting), then, after counting they are listed according increasing DCX+ cell densities. The kind of arrangement adopted is explained in each figure legend.

10.Reults (page 27 of PDF), regarding 'the amygdala/brain volume ratio was substantially similar in all species': The authors should consider the paper by Barger et al., 2007 (AMERICAN JOURNAL OF PHYSICAL ANTHROPOLOGY 134:392-403), where they say 'The human lateral nucleus was larger than predicted for an ape of human brain size and occupied the majority of the basolateral division, whereas the basal nucleus was the largest of the basolateral nuclei in all ape species.'

ANSWER: here we do not understand; the paper by Barger et al (2007) confirms that is the size of some subnuclei (those forming the basolateral complex) to vary across species (as confirmed by other papers cited across the manuscript), while the amygdala/brain ration (intended as the entire amygdala, not subnuclei) remains substantially unchanged.
The volumes we show in Fig. 8 are those referring to the whole amygdala volume across species. Hence, what stated by the Reviewer in this point agrees with our results and conclusions (accordingly, we cited this additional Reference).

11. Table 1 (in page 30): I suggest to use another format in form of Table, instead of a list a features. For example, for each of the listed findings, you can check if it is so in the different species examined (each species in a different column).

ANSWER: The Table has been changed, as suggested (apart from creating columns for each species, since these features were consistently shared by all species analysed, with the only exception of confocal analyses in chimpanzees).

Reviewer #3:

In the current manuscript Ghibaudi et al perform a comparative analysis of a immature neuron population in postnatal amygdala across a number of species and postnatal ages. Focusing on the basolateral complex, the authors identify several unique features of this neuron population including a much-increased density in larger brained gyrencephalic brains (including primates) and a broader topographic distribution across subnuclei of the basolateral complex than previously identified.
The work represents a unique and significant contribution to our understanding of the structure and organization of the amygdala. The presence of a population of immature

excitatory neurons in mature brain is of high interest to the neuroscience community in general. The unique approach here to evaluate this phenomenon across a wide phylogeny is interesting and could lend important insight to the functional importance of these neurons. Their observation that larger brained highly gyrencephalic mammals seem to contain a much larger pool in amygdala could relate to an increased in the functional demands of these regions in more complex brains. Given the amygdala's contribution to neurodevelopmental disorders such as autism spectrum disorder, this work may also have important implications to disease.

ANSWER: we thank the Reviewer for his/her thorough analysis of the entire manuscript and for overall appreciation of our work.

The work is of high quality and the manuscript is in general, well written. However, there are some general and specific concerns that should be addressed before acceptance. Most of the concerns have to do with the manner in which the data are presented. These are outlined below.

General Comment

Throughout the manuscript the authors use commas as decimal points and decimal points instead of commas for reporting numbers. Should this be reversed?

ANSWER: commas and decimal points have been reversed, both in the manuscript text and in tables of Supplementary material.

The authors use the term "coronal plan" throughout manuscript. Should be changed to "coronal plane"

ANSWER: the mistake has been corrected.

The authors argue that the relative numbers of scINs is stable in adult primates. But other groups using stereological and non-stereological approaches have shown stark decreases in DCX+ cell number in human paralaminar nucleus (Avillo et al., 2018, Sorrells, 2019) across adulthood. Because paralaminar is not well described across multiple species, they regard the basolateral complex in general and argue the distribution of DCX INs is more widespread in primates than just paralaminar nucleus. Perhaps the reduction in IN numbers in paralaminar previously described in human relates to migration of INs into the basolateral complex as these cells are presumably being integrated into circuitry? The manuscript could benefit from evidence (or lack thereof) of INs migrating into amygdala nuclei from their origin. Could the authors not address this with a more detailed topographic analysis?

ANSWER: in our previous version of the manuscript, we did not consider the possibility of immature cell migration for two reasons:

1) In all our analyses, including cell counting using Neurolucida (in which all DCX+ cells are visualized and recognized by the operator), we did not observe the typical alignment or prevalent orientation that usually characterizes the systems of migrating cells.
2) The occurrence of unipolar/bipolar DCX-positive cells is also detectable in most of immature cells in the cortex (cortical immature neurons); yet we know that such cortical cells are not migrating, since they have already reached their final location in layer II during embryogenesis and remain in place waiting for their maturation (data confirmed by many papers, including studies with the DCX-cre transgenic mouse). Hence, very likely, the unipolar/bipolar morphology is simply the most common morphology of immature neurons in their early stage of immaturity. We added such considerations in the text.

Apart from these theoretical considerations, the Referee's comment is right, and by following his/her suggestion, we performed an additional, detailed analysis to count the DCX+ cells with migratory morphology (unipolar/bipolar cells showing a migratory-like shape) and to investigate their main orientation of in all the animal species considered. To do this, we mapped the location of small cells with oval/fusiform cell soma and migratory morphology, as well as the orientation of their leading-like process, to investigate whether they can be considered oriented in a particular direction (similarly to what performed by Sorrells et al., 2019, in humans). In each species, the analysis was carried out in the anterior, middle, and posterior part of the amygdala. The procedure is explained in Methods.

The results of such analysis are now reported in the section Results and in Supplementary Figure 7. Only a very small percentage of the DCX+ cells display a migratory-like morphology (their number decreasing in gyrencephalic species, becoming negligible in the very large brains of chimpanzee and horse. Most importantly, they show a random orientation with no prevalent direction and can be found in all species within the nuclei of the basolateral complex (lateral, basal and accessory basal). Only in cat, sheep, horse and chimpanzee 1 or 2 cells were also found in the centromedial nuclei pointing outside the amygdala, while only in horse they can be also found in the cortical nucleus. Most of all, the DCX+ cells with migratory morphology were not found associated with specific amygdalar locations reminiscent of migratory streams.
These results confirm that the existence of specific migratory routes can be excluded. However, as suggested by Sorrells et al. (2019) in humans: "it is possible that some of these cells retain short-range migratory behavior for years to adjust the final location of their soma." We described the results of our additional analysis, and they were discussed to support our conclusion; however, a sentence to leave open the above-mentioned possibility was added.

It's somewhat surprising that the percentage of type 2 INs is so small in chimpanzee although they had by far the greatest densities of INs. Given that the progressive insertion of these INs into existing circuits is hypothesized as a strategy for plasticity in higher mammals you might expect to see many more of the more morphologically mature INs in chimpanzee or marmoset. It appears, however, that horse had the most. Can the authors comment on this. Is it reflective of the cognitive demand for amygdala in horse versus primate? As mentioned

above, it would be interesting to know the relative topographic location of Type 2 INs versus type 1 if they are in the process of maturing and integrating into mature circuitry.

ANSWER:
Concerning the topographical location of type 2 Vs. type 1 cells, we did not mention this aspect because they were randomly located. This information has been added both in figure legend and main text.
Overall, the distribution of type 1 and type 2 cells was quite similar in all species studied so far, even in the cerebral cortex (La Rosa et al., 2020b). The most logical explanation is that a slow process of maturation of some subpopulations of DCX+ cells is constantly present, regardless of the brain region, the species, or the age (for the latter, it has been clearly shown in the piriform cortex of transgenic mice; Benedetti et al., 2023). The fact that these immature neurons (mostly in their small morphology, highly immature state) remain very numerous at adult and old stages in gyrencephalic species (this happens also in the cortex) suggests they represent a reservoir of undifferentiated elements that not necessarily must be “used” being integrated. We do not have an explanation for this, but all evidence supports this fact. What appears clear is that even increasing or accelerating the maturation rate of the immature neurons, the number of type 2 cells does not change, they simply mature and disappear because they express no more DCX (evidence obtained in transgenic mice). We consider that finding more or less Type 2 cells in different species depends on (unknown) conditions linked to those animals investigated, not worthy of much interest.
It is difficult to speculate on this aspect at present. However, a comment has been added in the Discussion, leaving open the interpretation about the significance of the “reservoir” at advanced ages.

Results

"Characterization of the amygdala DCX+ neuronal population across mammals"
Paragraph 1, line 1-remove inappropriate "the"

ANSWER: “the” has been removed.

"Quantification of DCX+ neurons and dividing cells (cell densities) in the amygdala of different Mammals"
3rd paragraph, line 9 "plan" to "plane"

ANSWER: changed

"Topographical distribution of DCX+ neurons and Ki67+ dividing cells in the amygdala of mammals"
Last paragraph, changed "As showed..." to "As shown..."

ANSWER: corrected

Materials & Methods

All known ages should be reported for animals used.

ANSWER: All ages were already reported in Table S1 (in the Supplementary material; we fear that such material was not available to the Reviewers).

Figures

Figure 1
Pie chart-what does the relative size of each pie refer too. 2 pie pieces aren't labeled, what do these refer to. What do the red shapes refer to in the bottom part of the figure?

ANSWER:
- this is the mammal species pie chart, illustrating the distribution of extant and recently extinct mammal species across orders (based on Wilson, D. E. and D. M. Reeder eds. 2005. Mammal Species of the World a taxonomic and geographic reference third edition. Johns Hopkins University Press, Baltimore). We indicate the six orders, and eight species considered in the study. The explanation has been inserted in the Figure legend.
- the red shapes refer to the age ranges (from prepuberal to aged), to be used in plots of Figures 5 and 8.

I think the statement "...followed by staining of sections and segmentation of the subcortical region based on histology (B, bottom):" should read "...followed by staining of sections and segmentation of the subcortical region based on histology (C, bottom):"

ANSWER: YES. It has been corrected.

Figure 2
Worry that the labeling in Chimpanzee Amygdala includes off target. How is specificity evaluated in this species? Would be useful to see positive and negative control regions (eg SVZ labeling is in neurogenic niche or also has extra labeling) for chimpanzee.

ANSWER: We understand the worry of the Reviewer. Indeed, the number of DCX+ cells in the chimpanzee amygdala surprised us since the beginning, being really high (up to 7,000 average cells in single coronal sections – see line plot in Fig. 6A) forming a tightly packed network in the basolateral complex. As requested, we added controls, now showed in supplementary material (Fig S3). Yet, the SVZ was not used as control in chimpanzees since our animals are young adult and senior, and this neurogenic zone is exhausted very early (preliminary results from another study, in which we also noticed only a few DCX+ cells in the hippocampus). The SVZ was used for other species (see Fig. 4) to check specificity of the Santa Cruz anti-DCX antibody (used in this study for DCX detection). Another very good control for the Santa Cruz antibody (even in chimpanzee) is represented by the cerebral cortex layer II (piriform cortex;

wherein we know that typical DCX+ immature neurons are present in all species studied so far; see La Rosa et al., 2020 eLife). We inserted an image of DCX+ cells in the piriform cortex of one of the chimpanzees used in the study (aged group), now in a supplementary figure (Fig. S3).
In addition, we replicated the same result in the amygdala of chimpanzee by using a new antibody (Cell Signalling; now added in Table S2) that was made available only recently (image in Fig. S3). In our knowledge this antibody is now the only anti-DCX tool working well on chimpanzee brains, the Santa Cruz being out of commerce since several years (we had a large stock to be used in the comparative work in the amygdala replicating the same conditions used for the cortex).

Authors prior evaluation of antibody labeling (Ghibaudi et al, 2023) was on new world monkeys, not chimpanzee.

ANSWER: That technical study was performed in Turin by using confocal analyses. We are working on a new report including three primates (marmoset, macaque and chimpanzee) on which the new anti-DCX Cell Signalling antibody was tested successfully. At the time the technical work was published (2023) such antibody was still not available.

Figure 3
In legend, did you mean to use "douplets" or "doublets"

ANSWER: we mean “doublets”. It has been corrected.

Figure 5
Scale on Y axis of Figure 5b should be modified so the values and difference between species can be better appreciated

ANSWER: We made a line plot for Fig 5A with an increased scale, to better appreciate the interspecies differences (now Fig. 6A), yet the aim of Fig 5b is to show that values for Ki67 antigen (dividing cells), in contrast with DCX+ cells are flattened on lower levels in most species. In this case the interspecies differences are not important (apart from a more “proliferative tissue” in mouse and rabbit a very low values for other species, an aspect that is visible in the current plot), because this is the normal rate of oligodendrocyte progenitors, independent from the occurrence of immature neurons.

Scales for phylogenetic plots in Figure 5E a, b, c should have values and should be larger. Hard to see even when zoomed in

ANSWER: Indeed, the text was very small. It has been enlarged.

Figure 6

6A: The observations here are interesting. Seems in order to better visualize the comparison across species a normalized line plot would useful so all the species could be plotted on same graph and directly compared.

ANSWER: A line plot was produced, as suggested (now in Fig. 6A).

6B: similar thing could be useful here. Also, the Y axis values should be adjusted so that the graph values can be seen. For most of the plots it's difficult/imposible to view the values and therefore spatial distributions cannot be seen.

ANSWER: Same comment as the previous point: here the aim is to show that most of the Ki67+ cells (especially in gyrencephalic species and primates) are flattened on low levels and uniformly distributed in the entire length of the amygdala.
The original figure with histograms was maintained but placed in supplementary material, now Fig. S5).

Axes for plots should have values (eg slice umber, number of cells)

ANSWER: YES. We introduced the values of the axes on top left (A) and top right (B); there is no space enough to put them in each of the 12 plots.

Figure 7
Appropriate scale bars should be provided on representative images from each species.

ANSWER: Scale bars have been inserted.

Figure 8

For the graphs in A, B, C & D; It is very difficult to distinguish the age groups for given species using the current plot style. Data points associated each age goup should be gven different colors within each graph. For example, in A, for mouse the prepubertal are triangles but it's still difficult to separate them from the other three age groups. If each age group were different colors, it would be easier to distinguish. This should be applied to each of these graphs.

ANSWER: we reported higher magnifications of the plots A and B (now in Fig S6), by using different colors for ages, as suggested; the colors (different shades of green) are the same described in the color code of Fig. 1.

We maintained the plots in C and D, since these are just a confirmation (through analysis of our specimens) that total amygdala volume does increases with brain size but the amygdala/brain volume does not substantially change (as reported in literature).

The level of significance reported in each graph should be reported in the legend. Do 2 symbols (eg A, B; && vs &, D; ** vs *) indicate higher significance?

ANSWER: Yes, two symbols indicate higher level of significance (p<0.01). The level of significance is now reported in legends for Figure 5 and Figure 8.

Table 1
Table should be laid out correctly with, title, brief legend, defined rows and columns with headers

ANSWER: The Table has been changed, as suggested.

Table S2
RRID values should be provided for antibodies

ANSWER: We have now included the RRID for each antibody in Table S2.

Figure S1
Sheep posterior outline not numbered

ANSWER: YES (actually, it was the chimpanzee outline). Now numbered.
